# Exosomes: Implications in HIV-1 Pathogenesis

**DOI:** 10.3390/v7072810

**Published:** 2015-07-20

**Authors:** Marisa N. Madison, Chioma M. Okeoma

**Affiliations:** 1 Department of Microbiology, Carver College of Medicine, University of Iowa, Iowa City, IA 52242, USA; E-Mail: marisa-madison@uiowa.edu; 2 Interdisciplinary Program in Molecular and Cellular Biology, University of Iowa, Iowa City, IA 52242, USA

**Keywords:** extracellular vesicle, nanoparticle, exosome, semen, seminal plasma, HIV-1, murine AIDS

## Abstract

Exosomes are membranous nanovesicles of endocytic origin that carry host and pathogen derived genomic, proteomic, and lipid cargos. Exosomes are secreted by most cell types into the extracellular milieu and are subsequently internalized by recipient cells. Upon internalization, exosomes condition recipient cells by donating their cargos and/or activating various signal transduction pathways, consequently regulating physiological and pathophysiological processes. The role of exosomes in viral pathogenesis, especially human immunodeficiency virus type 1 [HIV-1] is beginning to unravel. Recent research reports suggest that exosomes from various sources play important but different roles in the pathogenesis of HIV-1. From these reports, it appears that the source of exosomes is the defining factor for the exosomal effect on HIV-1. In this review, we will describe how HIV-1 infection is modulated by exosomes and in turn how exosomes are targeted by HIV-1 factors. Finally, we will discuss potentially emerging therapeutic options based on exosomal cargos that may have promise in preventing HIV-1 transmission.

## 1. Introduction

Exosomes were discovered over 30 years ago when two different research groups led by Philip Stahl [1] and Rose Johnstone [2] independently described multivesicular late endosome back fusion to reticulocyte plasma membranes resulting in release of vesicular cargo, later termed exosomes [3]. Interest in exosomes was renewed in 1996 when exosomes secreted from B lymphocytes were shown to present major histocompatibility class II—antigen complexes to T lymphocytes [4]. Furthermore, in 2007, exosomes from human and murine mast cells were discovered to contain functional mRNA and miRNA that could be donated to recipient cells and translated into heterologous or homologous proteins [5]. Exosomes are membranous nanovesicles originating as a result of inward budding of endosomal membranes within the late endosomal compartment of a multitude of cell types [6,7,8]. Exosomes are released by many cell types [9] into the extracellular environment and are found in biological fluids [biofluids], notably, blood [10], urine [11], saliva [12], breast milk [13,14], and semen [8,15,16,17,18,19,20,21,22,23,24]. The molecular composition of exosomes closely follows that of the parental cells and varies between exosomes, and possibly between donors. Proteome and transcriptome studies have shed light into the cargo composition of exosomes from various sources. Functionally, exosomes have been implicated in modulation of host immune responses and microbial pathogenesis. In these roles, exosomes mediate intercellular communications [24,25,26,27], thereby providing the host with a mechanism to mount efficient immune responses against pathogens, including activation of antiviral pathways or transfer of antiviral factors between different cells [24,27,28,29]. In addition, exosomes can enable the virus to overcome host immunity by delivering viral antagonists or proviral/virulence factors to target cells [30,31].

The multifunctional way that exosomes affect the host microenvironment is evident in the study of exosomes and HIV-1. Exosomes from a range of cells mediate micro-environmental changes in target cells by inducing pro- or anti- viral responses [24,32,33,34,35] during HIV-1 infection. The ability of exosomes to inhibit or enhance HIV-1 infection depends on factors including but not limited to the infection status of the exosome producer cells, the source of the exosomes (biofluid or cell culture derived), and the type of producer and recipient cells involved in exosome donation and uptake. Therefore, the role of exosomes in HIV-1 pathogenesis may be significant and underappreciated.

## 2. Exosomes: Secreted Vesicles Loaded with Various Cargos

Exosomes are a class of extracellular vesicles (EV) generated by living mammalian cells (humans and animals) and microorganisms, including protozoa [36], fungi [36], and bacteria [37]. Other classes of EV include (I) apoptotic bodies that are generated by plasma membrane blebbing during cell death and (II) microvesicles [also known as microparticles or ectosomes] that are assembled at and are released from eukaryotic cellular plasma membranes via ectocytosis. Comparative analyses of various EV show that exosomal cargos are distinct from the cargos of other vesicles. Exosomal proteins (membrane and luminal) are derived from the cytosol, the endocytic compartments, or the plasma membrane [24,38] and are devoid of serum proteins and protein components of intracellular compartments, including, the endoplasmic-reticulum, Golgi-apparatus, mitochondria, or nucleus. Depending on the origin, exosomes generated by different cell types contain varying levels of endosome-associated proteins, such as Rab GTPase, SNAREs, Annexins, flotillin, and Tsg101 [39]. Also enriched in exosomes are tetraspanins; CD9, CD63, CD81, CD82, CD53 and CD37 [24,40,41], lipid rafts associated proteins [glycosylphosphatidylinositol-anchored proteins and flotillin) [42,43], cholesterol, sphingomyelin, ceramide and phosphatidylserine, but not lysobisphosphatidic acid (LBPA) that are present in ectosomes [44]. 

Aside from proteomics, various investigators have utilized different analytical techniques including unbiased deep sequencing and PCR assays to show that exosomes contain different RNA species, notably mRNA and miRNA. Also present in exosomes are small noncoding RNA species, including structural RNAs, tRNA fragments, vault RNA, Y RNA, repeat sequences, RNA transcripts overlapping with protein coding regions, and small interfering RNAs [24,25,45,46]. Interestingly, differences exist between exosomal RNA content and the RNA content of the originating cells [24,46]. The reason for such variation in RNA content is unknown. However, it is speculated that RNA molecules may be selectively incorporated into exosomes. Whether or not exosomal RNA molecules are directly enwrapped within the cytosolic lumen of exosomes or co-purified as component of RNA-protein complexes associated with exosomes is unclear. As a consequence, there exist inconsistencies in the RNA cargo of exosomes. Studies involving different RNA isolation approaches, sucrose flotation gradient, and resistance to RNase digestion could shed light on the true RNA composition of exosomes. With the high interest in exosome cargo composition, various approaches have been used to determine the cargo composition of different exosomes and the results are deposited at ExoCarta [47] and Vesiclepedia [48].

While it is clear that exosomes contain proteinaceous and genetic cargos, the molecular mechanisms governing cargo recruitment into exosomes as well as cargo release by exosomes are yet to be defined. However, it is believed that Endosomal Sorting Complex Required for Transport (ESCRT) family members known to play a role in the formation of intraluminal vesicles (ILV) are involved in exosome biogenesis. Out of the four ESCRT complexes, ESCRT-0 plays a role in ubiquitin-dependent cargo clustering. An ESCRT-0 member HRS was shown to be required for exosome secretion from various cancer cells [49,50,51]. ESCRT-I and ESCRT-II induce bud formation and the function of ESCRT-III involves vesicle scission. The ESCRT-1 associated protein TSG101 is critical in exosomal secretion. Studies have shown that reduction in TSG101 levels results in reduced exosome secretion from cancer cells [49,52]. Additionally implicated in exosome secretion are various isoforms of CHMP4 and ESCRT-III-associated protein, Alix, which when silenced, results in decreased exosome secretion [52]. 

Exosomes can also be secreted independently of the ESCRT machinery. It has been demonstrated that some cells depleted of key subunits of all four ESCRT proteins secrete exosmoses using ESCRT-independent mechanisms [53]. Exosome secretion in oligodendrocytes is facilitated by lipid ceramide independently of the ESCRT machinery [54]. Proteins of the tetraspanin family have been implicated in sorting cargos for exosome secretion. Tetraspanins are enriched in ILV/exosomes within late endosomes also known as multi vesicular bodies (MVB) [52]. The tetraspanin CD63 promotes endosomal sorting of melanosomal protein [55], while CD81 allows targeting of a variety of its ligands into exosomes [56].

Aside from tetraspanins, proteomic studies identified the RAB family of small GTPase proteins as exosomal cargo [38]. RAB proteins are involved in varied processes of vesicular trafficking [budding, mobility through cytoskeletal interaction, vesicle docking to target compartment] and membrane fusion and are therefore implicated in exosome release. For example, RAB11, RAB27a and RAB27b have been shown to control different steps of the exosome secretion pathway in different cells [57,58]. Screening and functional analysis of various RAB proteins following RNAi depletion, provided evidence that in cancer cells, loss of RAB5A, RAB9A, RAB2B, RAB27A, and RAB27B, efficiently decrease exosome secretion, while depletion of RAB11A or RAB7 does not affect exosome secretion [57]. 

The perceived involvement of the ESCRT machinery and the RAB proteins in exosome biogenesis has led to the use of ESCRT and RAB inhibition as an acceptable method for perturbing exosome secretion. However, given the role of ESCRT and ESCRT-associated proteins in plasma membrane-dependent budding of enveloped viruses [59,60,61], cytokinesis, and membrane repair, as well as the role of RAB proteins in secretion of other soluble factors; inhibition of ESCRT and RAB pathways may modulate host cellular functions unrelated to exosome formation/secretion or may alter the cargo composition of released exosomes. Therefore, caution should be applied while analyzing/using exosomes generated following such treatments. 

## 3. HIV-1 Exploits Exosomal Machinery: HIV-1 RNA and Protein Trafficking via Exosomes

The biogenesis of exosomes and the cell types that released the exosomes determines exosomal content. The study of exosomes secreted from HIV-1 infected cells is complicated by the fact that exosomes have many features in common with HIV-1 including biophysical/molecular properties, biogenesis and uptake mechanisms. The density of exosomes range from 1.13 to 1.21 g·mL^−1^ [38], while HIV-1 density ranges from 1.16 to 1.18 g·mL^−1^ [62]. In size, HIV-1 is slightly larger than exosomes, with the diameter of the virus ranging from 100–120 nm compared to 40–100 nm diameter of exosomes [63]. In addition, it is thought that HIV-1 can be generated by the same pathway of exosome biogenesis [64]. Budding of HIV-1 involves interaction with a number of cellular factors, such as TSG101 and Alix that are also involved in exosome biogenesis [65]. The convergence of HIV-1 and exosome biogenesis suggests that HIV-1 products, including RNA and proteins may be encased within exosomes or contaminate exosome preparations from HIV-1 infected fluids. However, rigorous purification strategies, including iodixanol density gradients and immuno affinity approaches are capable of separating HIV-1 particles from exosomes [63,66]. The striking similarities between the biogenesis of exosomes and enveloped viruses in general and HIV-1 in particular, led to the Trojan exosome hypothesis of HIV-1 assembly and cell to cell spread [67], which proposes that HIV-1 evolved to utilize exosome biogenesis and uptake pathways for the formation of infectious virus and for env-independent virus entry respectively.

In support of the Trojan exosome hypothesis, HIV-1 recruits components of the host ESCRT machinery to the sight of viral budding and the virus shares some similarities with shedding microvesicles/ectosomes. In addition, the interaction between HIV-1 Gag protein and tetraspanins [68] suggest that HIV-1 may utilize lipid raft micro-domains rich in tetraspanins for virus assembly. Indeed, CD81 and CD63 found on the surface of exosomes are known to be involved in HIV-1 budding, cell-to-cell spread, and infectivity [68,69,70,71]. Moreover, HIV-1 and exosomes express sialyllactose-containing gangliosides that can interact with sialic-acid-binding immunoglobulin-like lectins (Siglecs) present in mature dendritic cells (mDCs) [72]. It has been shown that Siglec-1 interacts with sialyllactose-containing ganglioside on HIV-1 [72] and exosomes [72,73], and promotes mDC capture and storage of both HIV-1 and exosomes in mDCs, thus facilitating *trans* infection of T cell by mDCs [74,75]. It is therefore speculated that exosome competition for binding to Siglec-1 may result in diminished *trans* HIV-1 infection. While the involvement of the exosomal pathway in HIV-1 entry and budding is thought provoking, the Trojan exosome hypothesis came to light during a time when terminology was limited with regard to extracellular vesicles and when the field of exosome research was just beginning to expand. Thus, the Trojan exosome hypothesis is a misnomer because HIV-1 is more aptly compared to ectosomes/microvesicles as evidence suggests that HIV-1 buds from the plasma membrane, the budding site of ectosomes and not from internal MVB membranes, which largely represents the exosome budding site.

Host-derived exosomal RNA and protein cargos are trafficked from one cell type to the other. Some of these cargos are common to all exosomes while others are specific to the producer cells from which they are secreted. Some cargos present in exosomes isolated from HIV-1 infected cells are viral components, suggesting a role for exosomes in facilitating viral evasion of host immunity. There is evidence that HIV-1 infection or transduction of cells with the HIV-1 accessory protein, Nef, increases the cellular release of exosomes [34,76,77,78]. Nef interacts with intracellular vesicular sorting and trafficking pathways and directs MHC-I [79,80] and CD4 [80,81] to MVBs for lysosomal degradation of MHC-I [80,82] and CD4 [80,83,84,85]. In addition, Nef is sequestered within exosomes released from cells *in vitro* and within blood plasma-derived exosomes from HIV-1 seropositive individuals [34,76,77,78,86]. Although the mechanism of Nef association with exosomes is not fully understood, Nef is encased in exosomes by anchoring to exosome lipid raft micro-domains. This process involves Nef’s N-terminal myristoylation and amino acids within the alpha helix 1.

Other viral proteins have also been shown to be targeted to exosomes. HIV-1 Gag is targeted to exosomes via Gag higher order oligomerization [64]. Aside from viral proteins, evidence indicates that genomic unspliced HIV-1 RNA is encased in exosomes isolated from chronically infected U937 cells [87]. The encasement of HIV-1 RNA into exosomes is mediated by the 5′ end of Gag p17 matrix open reading frame. Unlike Pegivirus RNA encased in exosomes [88], HIV-1 RNA encased in exosomes is not infectious. However, increased association of HIV-1 RNA with exosomes correlates with decreased levels of HIV-1 RNA packaging in viral particles [87]. In addition to HIV-1 RNA, HIV-1-derived miRNAs including vmiRTAR [89], vmiR88 [90], and vmiR99 [90] are packaged into exosomes derived from HIV-1-infected cultures and blood of HIV-1-seropositive patients. Exosomal vmiRTAR reduces expression of Bim and Cdk9 proteins in target cells resulting in decreased apoptosis. Since packaging of HIV-1 RNA into exosomes reduces the available viral RNA for particle assembly, it is possible that the host utilizes delivery of genomic HIV-1 RNA to exosomes as part of the defense mechanism for elimination of viral genomes. If this is proven to be true, the consequence could be host cellular modification of the HIV-1 genome to be preferentially diverted to exosomes routed for lysosomal degradation. However, HIV-1-derived vmiRTAR encased within exosomes may function to promote HIV-1 infection and increase disease pathogenesis.

## 4. Exosomes Released by HIV-1-Infected Cultured Cells Contain HIV-1-Derived Virulence Factors and Influence Host Cell Infection

**Figure 1:**
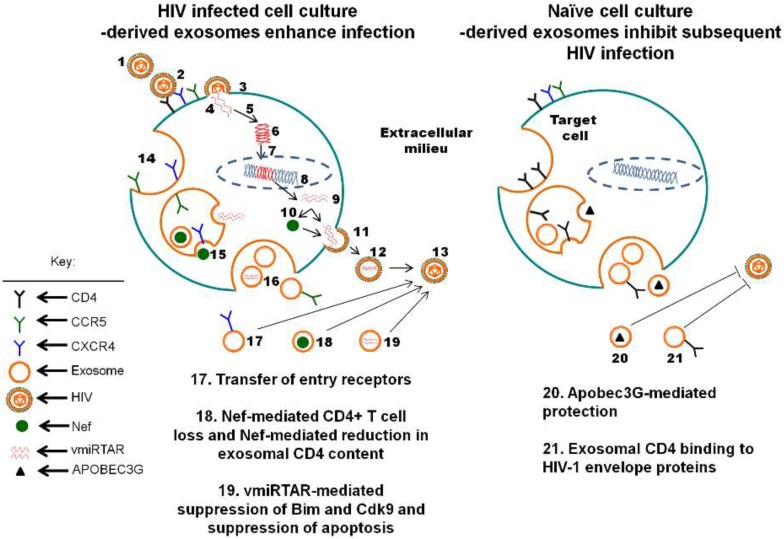
Cell culture derived exosomes modulate HIV-1 infection: (**1**) Cell-free HIV-1 virions bind host cells via viral envelope (Env) to (**2**) CD4 on target cell plasma membrane followed by binding to a viral co-receptor, CXCR4 or CCR5; (**3**) Co-receptor binding initiates viral fusion, whereupon viral contents including (**4**) single stranded viral genomic RNA (gRNA); reverse transcriptase (RT) and integrase (IN] are emptied into the cellular cytoplasm. Viral gRNA is (**5**) reverse transcribed by HIV-1 reverse transcriptase (RT) into (**6**) double stranded viral copy DNA (HIV-1 DNA), which is incorporated by (**7**) viral IN enzyme as (**8**) proviral DNA within human genomic DNA (gDNA). The provirus is transcribed by host cellular machinery into (**9**) nascent viral RNA, which is translated by host cellular machinery into (**10**) HIV-1 proteins, including Nef. Viral proteins are assembled with nascent viral RNA including HIV TAR RNA sequences (vmiRTAR) into (**11**) budding progeny virions. During the budding process viral protease initiates cleavage of viral proteins in budding progeny. Budding progeny break free from the cell as (**12**) immature progeny within the extracellular milieu, which undergo maturation with the completion of viral protease-mediated processing into (**13**) mature progeny capable of propagating productive HIV-1 infection in neighboring and distal cells; (**14**) Inward budding of the cellular plasma membrane during endocytosis generates endosomes; (**15**) Inward budding of the endosome membrane generates exosomes with cellular cytosol derived proteins and nucleotides sequestered within the exosome lumen, including HIV Nef and vmiRTAR; (**16**) Back fusion of the endosome containing exosomes (multivesicular body) to the cellular plasma membrane releases exosomes into the extracellular milieu. Exosomes derived from HIV-1 infected cell cultures may enhance infection through the following mechanisms: (**17**) transferring viral binding and entry receptors to HIV-1 susceptible cells, thereby increasing the expression of viral binding sites on the surface of the host cellular plasma membrane. (**18**) Nef-mediated internalization and degradation of CD4 molecules and Nef-mediated reduction of CD4 expression on the surface of exosomes and (**19**) suppression of Bim and Cdk9 expression and apoptosis by viral miRNA generated from vmiRTAR. The protective roles of cell culture derived exosomes from uninfected cells may be mediated by: (**20**) Apobec3G enwrapped within exosomes and (**21**) exosomal CD4 binding to HIV-1 Env.

The function of exosomes in HIV-1 pathogenesis is beginning to emerge. Accumulating data reveal that exosomes released from HIV-1 infected cells have distinct functions from exosomes released from uninfected cells and biofluid. Exosomes released from infected cells have been shown to mostly enhance infection or function as immune decoys [Figure 1]. In contrast, exosomes from uninfected cells or from HIV-1-seronegative biofluid have protective properties. For example, exosomes from HIV-1 infected macrophages sequester HIV-1 particles and have been shown to facilitate viral transfer to uninfected cells [91]. However, Kadiu and colleagues showed that HIV-1 sequestered by exosomes isolated from infected macrophages are not capable of CD4-independent infection [91]. The inability of macrophage exosomes to mediate CD4-independent HIV-1 infection support the notion that HIV-1-loaded exosomes utilize a different route such as clathrin-mediated endocytosis to gain entry into host cells [91]. Whether or not exosomes secreted from macrophages contain CD4 is unclear. However, it has been demonstrated that various types of EV mediate the transfer of HIV-1 co-receptors CCR5 and CXCR4 to co-receptor-null cells [92,93]. CCR5+ microvesicles released by CCR5+ Chinese hamster ovary cells and peripheral blood mononuclear cells transferred to CCR5− cells rendered these cells CCR5+. The transfer of CCR5 to CCR5-deficient peripheral blood mononuclear cells allowed infection of these cells with R5 tropic HIV-1 [92]. Similarly, transfer of microvesicle-derived CXCR4 to cells that lack the expression resulted in HIV-1 infection of such cells [93]. These results point to the possibility that exosomes may facilitate expression of HIV-1 entry receptors in an otherwise negative background, thus modifying viral tropism. Indeed, HIV-1 infections of cells that do not express HIV-1-entry receptors/co-receptors have been demonstrated without a clear understanding of the mechanisms governing such observations [92,93,94,95]. Furthermore, CD4 has been found on exosomes and in endocytic compartments where exosome biogenesis occurs [33,81,83,84,85,96]. It is possible that exosomes and other related EV may play a role in modulating infection of cells that do not constitutively express HIV-1 receptors and/or co-receptors, given the accumulating evidence that these structures can transfer HIV-1 entry receptors between cells.

Aside from the role of exosomes in facilitating HIV-1 infection by transfer of the viral entry receptors, exosome-mediated transfer of viral proteins, such as HIV-1 Nef to host cells can modulate infection. Nef reprograms endocytosis, cytoskeletal rearrangement and organelle trafficking [97]. These Nef-mediated events increase the number of endosomes, lysosomes, MVBs, HIV-1, and exosomes released from HIV-1 infected and bystander cells [78,98,99,100]. Exosomal-Nef may play a role in HIV-1 pathogenesis by promoting CD4+ T cell depletion in HIV-1-seropositive individuals. The evidence for this assumption comes from the observation that Nef-loaded exosomes condition target cells, such as CD4+ T cells by inducing apoptosis [34,86]. Although Nef increases exosome secretion, it was shown that Nef has the ability to reduce the amounts of both CD4 and MHC-I molecules loaded into exosomes [33]. This selective and paradoxical role of Nef suggests that Nef-loaded exosomes play complex roles in HIV-1 infection and pathogenesis. Indeed, CD4-loaded exosomes efficiently inhibit HIV-1 infection, while CD4-depleted exosomes isolated from CD4+ T cells expressing Nef have a reduced capacity to inhibit HIV-1 infection [33]. These observations suggest that CD4-loaded exosomes may provide inhibitory effects on HIV-1 infectivity by virtue of exosomal CD4 binding to HIV-1 envelope proteins. However, the ability of Nef to selectively eliminate CD4 from exosomes suggests yet another mechanism by which Nef promotes HIV-1 infection. Indeed, Nef induces uploading of ADAM17 in exosomes from HIV-1 infected cells. While resting CD4+ T lymphocytes are refractory to HIV-1 infection, exposure to Nef and ADAM17 containing exosomes renders these cells permissive to HIV-1 infection [30].

Contrary to evidence demonstrating that HIV-1 mediated hijacking of exosomal machinery enhances infection and thwarts host defense, there is evidence in support of the role of exosomes in inhibiting viral infections. It has been shown that exosomes and related EV have the capability to sample viral antigens and present these antigens to the relevant immune cells, with resultant stimulation of antiviral response in the host [4,8,101]. Exosome-like structures secreted by airway epithelial cells in culture possess anti-viral properties against influenza virus [102]. With respect to HIV-1, exosomes derived from the T lymphocyte H9 cell line possess anti-HIV-1 activity that was attributed to the presence of the cytidine deaminase Apolipoprotein B mRNA-editing, enzyme-catalytic, polypeptide-like 3G (Apobec3G) enwrapped in the exosomes [103]. Since Apobec3G is an anti-HIV-1 host factor, this finding suggest that exosomes may contribute to host innate immune defense mechanisms by enwrapping and transferring anti-viral factors to target cells. 

## 5. The Function of Biofluid Exosomes is Distinct from Cell Culture-Derived Exosomes: Intrinsic Protection from HIV-1

**Figure 2:**
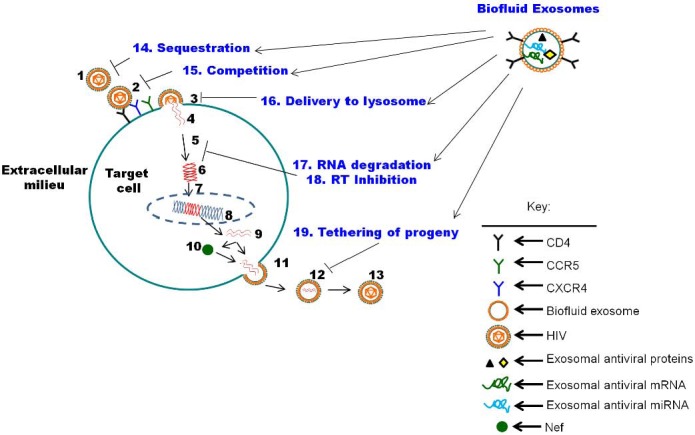
Model of biofluid exosome antiviral functions within the HIV-1 life cycle: (**1**–**13**) HIV-1 life cycle as defined in Figure 1 legend. Biofluid exosomes may interact with free HIV-1 and (**14**) sequester free viral particles within exosome aggregates thereby inhibiting HIV-1 infection by preventing virus from binding to target cells. Biofluid exosomes that present CD4 molecules on the exosome surface may (**15**) compete for viral Env binding to CD4 on the host cell plasma membrane, thereby inhibiting HIV-1 infection by preventing virus from binding to target cells. Competition may also occur via binding of exosome ligands/receptors to other receptors/ligands on the viral envelope (e.g., phosphatidylserine/annexin interaction). Exosomes are taken up by cells via direct fusion with the plasma membrane or by endocytosis into endosomes that may subsequently fuse with lysosomes. Exosomal interaction with HIV-1 may lead to entry of the virus into the cell via endocytosis, leading to (**16**) exosomal delivery of virus into lysosomes for degradation, subsequently inhibiting HIV-1 infection. (**17**) HIV-1 gRNA may be degraded or otherwise rendered non-functional by exosomal antiviral proteins including Apobec3g, or following translation of exosomal antiviral mRNA into antiviral proteins including Apobec3g, or by antiviral exosomal miRNA. Exosomal antiviral protein, mRNA or miRNA may (**18**) inhibit viral RT and reverse transcription processes by blocking RT activity, blocking RT binding to RNA or facilitating degradation of RT. In the event that exosomes fuse with the cellular plasma membrane, they may enrich the cell surface with proteins that may function to (**19**) tether budding progeny virions to the plasma membrane (e.g., BST-2/tetherin, PS/Annexin), preventing them from diffusing into the extracellular milieu and subsequently preventing HIV-1 propagation.

Unlike biofluid exosomes generated by a multitude of cell types *in vivo*, cell culture exosomes originate from homogeneous and often times transformed cells that hardly recapitulate the *in vivo* microenvironment. Indeed, exosomes derived from tumor cells cultured *in vitro* differ from exosomes isolated from tumor cells grown *in vivo* [104]. The anti-viral properties of cell culture derived exosomes pales in comparison to that observed in biofluid derived exosomes. Early studies on exosome functions in the late 1990 and early 2000 revealed that constituents of human semen could impair bacterial growth and can neutralize measles virus [105,106], illustrating the potential protective properties of semen. Thus far, exosomes isolated from two types of biofluid from HIV-1-seronegative donors have been shown to inhibit HIV-1 infection (Figure 2). Exosomes purified from breast milk inhibit HIV-1 *trans* infection mediated by dendritic cells (DC) by binding DC-SIGN and blocking DC-mediated viral transfer to CD+ T cells [29]. The binding of DC-SIGN by milk exosomes signify potential competition between milk exosomes and HIV-1 for binding to DC-SIGN.

In sharp contrast to milk exosomes, blood-derived exosomes from plasma of HIV-1-seronegative donors do not inhibit HIV-1 infection [29]. The inability of blood-derived exosome to inhibit HIV-1 infection was later confirmed in a different study by our research group [24]. However, we discovered that exosomes purified from semen of HIV-1-seronegative donors inhibit infection of various cell types by different strains of HIV-1 (Lab adapted and transmitted founder viruses) irrespective of co-receptor usage [24]. Internalization of semen-derived exosomes into target cells, namely human vaginal epithelial cells occurs mostly by endocytosis and partly by direct fusion with the cellular plasma membrane [27]. Upon internalization, semen-derived exosomes potently block cell to cell and *trans* HIV-1 infection from vaginal epithelial cells to monocytic and lymphocytic cell lineages, as well as peripheral blood leukocytes (PBLs) [27]. The anti-viral activity of semen-derived exosomes extends to other retroviruses and was observed *in vivo*. Semen-derived exosomes block intravaginal replication of murine AIDS (mAIDS) virus complex and restrict lymphatic mAIDS dissemination in mice [27]. While semen-derived exosomes potently block HIV-1 infection, both semen and blood-derived exosomes have no effect on replication of Herpes simplex virus (HSV) types 1 and 2 [24]. These observations signify that the antiviral activity of semen-derived exosomes is specific for HIV-1 and murine retroviruses. Although the exact mechanism of action of semen-derived exosomes is yet to be determined, we found that semen-derived exosomes exert a post-entry block on HIV-1 replication by orchestrating deleterious effects on virion-associated reverse transcriptase (RT) activity, thereby impairing particle infectivity [24]. While semen-derived exosomes inhibit viral RT activity, viral progeny generated in the presence of these exosomes have equivalent amount of p24 but diminished viral RNA (Gag) compared to progeny generated in the absence of semen-derived exosomes. It is surprising that the same amount of p24 is detected by ELISA and immunoblot analysis of HIV-1 progeny generated in the presence or absence of semen-derived exosomes, yet the fitness of the progeny generated in the presence of semen-derived exosomes is ablated. Intact progeny p24 in the face of diminished Gag RNA and reduced RT activity indicates that either (i) steady-state p24 from incoming virus is sufficient for incorporation into progeny virions; (ii) the threshold of Gag mRNA needed to generate p24 CA protein is established and, thus, p24 packaging into progeny is unaffected by semen-derived exosomes or (iii) p24 can be incorporated into exosomes as previously observed [64].

HIV-1 reverse transcription is integral to synthesis of nascent viral copy DNA required for establishing productive infection and semen-derived exosomes impair this process. Therefore, it appears that semen-derived exosomes have early post entry effects on HIV-1. If the effect of semen-derived exosomes on HIV-1 is solely on the RT process, then semen-derived exosome-mediated deleterious effects on HIV-1 observed at later time points past reverse transcription may be downstream effects of a cascade of events initiated during reverse transcription. It is also possible that semen-derived exosomes may affect HIV-1 at different stages in the viral life cycle. Further studies are needed to investigate these propositions. Additionally, additional studies are required to understand the effect of breast milk and semen-derived exosomes purified from HIV-1-seropositive donors on virus replication.

Not only do semen-derived exosomes have innate antiviral properties, these exosomes have natural anti-inflammatory functions. Various investigators implicated semen and its constituents in varied processes relating to spermatozoa function and protection from the female reproductive tract (FRT) immune responses [106,107,108,109,110,111,112,113,114,115,116,117,118,119,120,121,122,123]. Semen is thought to have both immuno-stimulatory and -suppressive properties. The cell-free component of semen, seminal plasma, contains signaling molecules (cytokines, chemokines, and prostaglandins). Interaction between these molecules and the female genital epithelial cells may induce an inflammatory cascade that includes recruitment and activation of cells. In contrast, exosomes in semen are speculated to be immunosuppressive [124]. Such speculation was later supported by a preliminary study from the Hladik lab showing that exposure of cells (DCs and PBMCs) to semen-derived exosomes results in impaired production of TNFα and IFNγ, co-stimulatory capacity of antigen-presenting cells, and antigen-specific T cell responses [125]. While immunosuppression mediated by semen-derived exosomes may exacerbate some disease outcomes; for viruses that thrive in an immune activated state, such as HIV-1, the anti-inflammatory function of semen-derived exosomes may be beneficial to the host. For example, semen-derived exosomes could suppress HIV-1-induced inflammation, cell activation, cytokine/chemokine synthesis, and immune cell homing to the site of infection. All of these have the potential to diminish virus spread, especially in the mucosa, thereby preventing sexual transmission of HIV-1.

## 6. Exosome-Associated HIV-1 Host Restriction Factors, Cytokines, and miRNAs

Exosomes exert their biological functions by direct interactions with recipient cells, or through the transfer of their cargo following membrane fusion with or endocytosis into target cells. Because various molecules are recruited into exosomes, it is has been suggested that exosomes can simultaneously transfer multiple effector molecules to target cells. This unique ability makes exosomes superior vehicles for cargo shuttling compared to other classical secretion mechanisms such as those used by cytokines, chemokines and hormones. Various reports have shown that exosomes from different sources can transfer pro- and anti- viral factors to target cells with resultant modulation of host cell responses [28,89,103]. Exosomal bio-physical and -chemical properties, cargo composition and functions differ depending on a range of factors including but not limited to the source of the exosomes and infection status of donors. For cell culture derived exosomes, variability in morphology, size and composition could be managed to an extent. However, as biofluid exosomes are generated from a diverse array of cell types within the host, it is plausible that donor dependent variability affects the cargo composition of exosomes isolated from the same biofluid but from different individuals; although, the antiviral activity of semen-derived exosomes from different donors is not significantly different [24,27]. Given the anti-HIV-1 activity of exosomes from milk and semen, it is conceivable that these exosomes are loaded with molecules that are known to restrict HIV-1 infection. It is also possible that exosomes from these sources elicit signaling events that consequently block HIV-1 infection. Indeed, semen-derived exosomes have been shown to be enriched in mRNA encoding an array of host restriction factors (HRF) including Apobec3 family members and BST-2/tetherin [24]. Donor variability was observed in some Apobec3 and BST-2 mRNA. Apobec3C, Apobec3D/E, Apobec3F, and Apobec3G mRNA were uniformly present in semen-derived exosomes from all donors examined. However, donor variability was observed in mRNA expression of Apobec3A, Apobec3B, Apobec3H and BST-2 in semen-derived exosomes. While semen-derived exosomes uniformly contain Apobec3C, Apobec3D/E, Apobec3F, and Apobec3G mRNA, blood-derived exosomes from all donors examined were completely devoid of Apobec3B, Apobec3C, Apobec3D/E, Apobec3H and BST-2 mRNA. While these observations are interesting, it is cautioned that these exosomes were not autologous. Therefore, it is necessary to profile HRF cargos from autologous semen and blood -derived exosomes during cargo composition and comparison studies. 

Additionally, the mRNA cargo of human semen-derived exosomes is transferable to mice. Apobec3G mRNA in exosomes isolated from human semen was transferred to mice following intravaginal inoculation of mice with these exosomes. In these mice, human Apobec3G mRNA was detected in vaginal cells [27]. This finding supports the premise that semen-derived exosomes transfer genetic material between heterologous species. Interestingly, not all HRF mRNA present in semen-derived exosomes were transferred to murine vaginal cells, suggesting the involvement of a possible specific requirement for heterologous cargo shuttling. It is unclear whether any of the HRFs contained in semen-derived exosomes participate in HIV-1 inhibition. Because HIV-1 counteracts the activity of the various HRFs loaded into exosomes, it is of interest to know whether differences exists in HRF composition of semen-derived exosomes derived from HIV-1-seronegative and HIV-1-seropositive donors. Going by current literature that showed that exosomes protect Apobec3G from HIV-1 Vif [103], it is speculated that HRF repertoire in exosomes from HIV-1-seropositive donors will be intact. 

Aside from the exosomal HRF mRNA cargo, exosomes isolated from blood of HIV-1-seropositive donors are enriched with proinflammatory cytokines and chemokines compared to seronegative plasma controls. In addition, cell surface expression of CD38 activation marker is increased on naive and central memory CD4+ and CD8+ T-cells in the presence of HIV-1-seropositive blood-derived exosomes compared to HIV-1-seronegative exosomes [126]. Therefore, it is possible that unlike semen-derived exosomes which have immunosuppressive properties, blood-derived exosomes may have immunostimulatory and cell activation effects. The presence of inflammatory molecules in blood-derived exosomes from HIV-1-seropositive individuals may facilitate HIV-1 propagation in the host by promoting inflammatory response, immune activation, chemotactic signaling, and massive supply of HIV-1-susceptible leukocytes to sites of infection. Indeed, we and others have observed that exosomes purified from HIV-1-seronegative blood donors have no effect on HIV-1 infectivity *in vitro* [24,29]. The role of exosomes from HIV-1-seropositive donors on HIV-1 infection of target cells is yet to be determined.

Additionally present in exosomes are miRNAs that have been shown to be protective against HIV-1. These include miR-28 [127,128], miR-29a [129,130], miR-29b [129], miR-125b [128,131], miR-149 [129], miR-150 [127,128], miR-198 [132], miR-223 [127], miR-324 [129], miR-378 [129], and miR-382 [127]. The level and roles of these miRNAs in the different types of exosomes are undetermined. For example, miR-382 is an anti-HIV-1 miRNA [127] that is packaged into semen [25,133] and blood [133] exosomes. Because semen-derived exosomes inhibit HIV-1 infection [24,27] but blood-derived exosomes do not [24,29], it appears that exosome associated miR-382 may not function as anti-HIV-1. If this logic is proven to be true, then anti-HIV-1 miRNA targeted to exosomes may have different functions unrelated to HIV-1. In addition, HIV-1-derived miRNA, including vmiRTAR [89], vmiR88 [90], and vmiR99 [90] are present in exosomes isolated from HIV-1 infected cultured cells and blood of HIV-1-seropositive patients. While vmiRTAR enhanced infection, vmiR88, and vmiR99 may function as chronic immune activators. The summary of HIV-1-relevant miRNA found in exosomes is presented in Table 1.

**Table 1:** HIV-1-relevant miRNA found in exosomes.

miRNA	Exosome Source	References
miR-28	Dendritic cells, J77 T cells, semen, blood	[133,134]
miR-29a	Breast milk, semen, dendritic cells	[25,133]
miR-29b	Semen, blood	[25,133]
miR-125b	Dendritic cells semen, blood	[25,133,134]
miR-149	Breast milk, semen dendritic cells, blood	[25,133]
miR-150	J77 T cells, breast milk, blood, semen	[133]
miR-198	Dendritic cells	[133]
miR-223	Dendritic cells, serum, breast milk, blood, semen	[133,134]
miR-324	Semen, blood, dendritic cells,	[25,133]
miR-378	Breast milk, semen, blood, dendritic cells,	[25,133]
miR-382	Semen, blood	[25,133]
vmiR88	Primary alveolar macrophages, serum	[90]
vmiR99	Primary alveolar macrophages, serum	[90]
vmiRTAR	J1.1 cells	[89]

## 7. Exosomes and Their Promise for Diagnostic and Therapeutic Applications

As stated earlier, exosomes loaded with host or pathogen derived RNA molecules, proteins and lipids are released by cells into their surrounding environment. The presence of large numbers of exosomes containing various molecules in several biofluids indicate that (i) *in vivo*, host cells can tolerate exosomes and their contents; (ii) exosomes serve as a vehicle to shuttle RNA molecules, proteins and lipids between cells over short and long distances; and (iii) the host could use exosomes as a channel for elimination of antigens. For instance, cancer patients have high concentrations of exosomes loaded with tumor-specific molecules. In these patients, exosomes can be used and is being evaluated for their potential use as cancer biomarkers. As biofluid exosomes carry and transport specific proteins and various RNA species derived from multiple organs, biofluid exosomes may be associated with a given disease or carry protective molecules [24,27,29,135]. 

The use of exosomes as biomarkers for therapeutic purposes for cancer has gathered momentum. However, the field of HIV-1 study is still in its infancy with regards to exosomes. Exosomes could potentially be used as biomarkers and as therapeutic tools for HIV-1. The detection of exosomes in blood of HIV-1-seropositive patients and the identification of HIV-1 proteins and RNAs in exosomes isolated from the blood of HIV-1-seropositive patients emphasizes the potential utility of exosomes and their contents as biomarkers for HIV-1. The demonstrations that HIV-1 RNA and protein are packaged into blood-derived exosomes suggest that in HIV-1-seropositive people, the body could clear viral factors by secreting them into exosomes. Blood-derived exosomes could therefore be harnessed as diagnostic and prognostic HIV-1 biomarkers by (i) stimulating the release of exosomes containing the HIV-1 genome to rid the body of viral factors; and (ii) efficiently targeting the HIV-1 genome to exosomes that have been engineered for efficient degradation. The presence of the HIV-1 genome in blood-derived exosomes could also be used as a tool to assess the efficacy of treatment. This could be done by evaluating the presence of specific viral molecules in exosomes as a means of assessing treatment efficacy with respect to suppression of viral load or disease progression.

**Figure 3:**
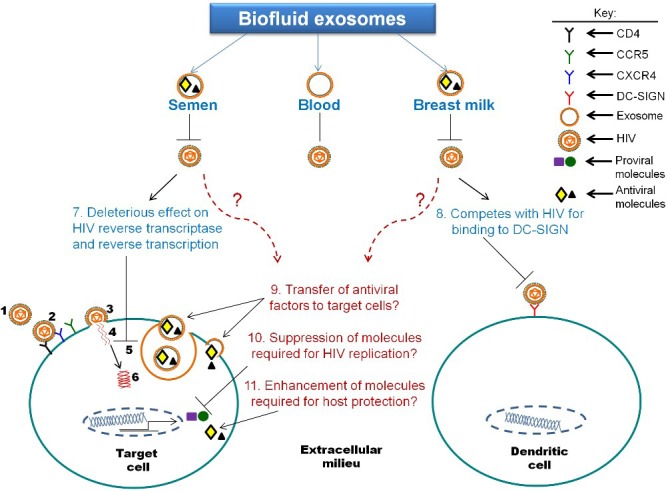
Biofluid derived exosomes modulate HIV-1 infection: (**1**–**6**) HIV-1 life cycle as defined in Figure 1 legend. Exosomes derived from human semen inhibit direct, *trans* and cell-to-cell transmission of HIV in human PBL, T cells and monocytes by (**7**) mediating deleterious effects on HIV reverse transcriptase and reverse transcription processes. Exosomes derived from human blood plasma or blood serum have no effect on HIV infectivity. Exosomes derived from human breast milk inhibit cell-to-cell transmission of HIV from monocyte-derived dendritic cells (MDDC) to CD4+ T cells by (**8**) competing for binding of HIV to DC-SIGN on MDDC. Biofluid derived exosomes may also inhibit HIV infectivity by (**9**) exosomal donation or transfer of antiviral cargo to recipient cells; (**10**) inhibition of cellular signaling or molecules required for HIV replication or (**11**) induction of cellular signaling or donation of exosomal factors resulting in enhancement of expression of molecules responsible for host protection against HIV.

Based on recent observations that exosomes derived from human breast milk [29] and from human semen [24,27] control HIV-1 infection of cells in culture (Figure 3), the anti-HIV effectors in biofluid exosomes or biofluid exosome based synthetic nanoparticle delivery systems can potentially be used as therapeutics in HIV-1 prevention and/or treatment. Furthermore, exosomes derived from human semen inhibit intravaginal replication of mAIDS virus when they were administered into the mouse vagina simultaneously with the virus [27] as would occur in human vaginal mucosa exposed to semen of HIV-1-seropositive individuals during sexual transmission. Systemic virus spread was reduced in mice that received the virus/exosome complex and plasma viremia was decreased. These observations suggest a positive therapeutic effect of semen-derived exosomes on mucosal HIV-1 transmission and systemic virus dissemination [27]. It is tempting to speculate that milk and semen-derived exosomes are endowed with intrinsic protective properties that limit the vertical [milk-borne) and horizontal (sexual) transmission of HIV-1. The beneficial roles of these exosomes are known but the mediators have yet to be identified. Upon identification of the mediators, several potential approaches for their use in therapeutic applications for HIV-1 include (i) engineering exosomes containing specific protective molecules; (ii) designing small molecule drug exosome mimics and delivering them to the targets, and (iii) delivery of semen-derived exosomes themselves to the mucosa as natural carriers of anti-HIV-1 molecules.

Furthermore, nanoparticle delivery systems mimicking semen-derived exosomes may have significant contribution in vaginal mucosal drug delivery based on the fact that semen-derived exosomes are more efficiently internalized than blood-derived exosomes and at least one cationic liposome formulation [27]. It is also plausible that nanoparticle delivery systems mimicking semen-derived exosomes may serve as a better delivery system because semen-derived exosomes can naturally overcome mucosal barriers that other artificially created vesicles are unable to surmount.

## 8. Conclusions and Perspectives

In order to achieve an HIV-1/AIDS-free world, we are in need of discovery and design of efficacious and affordable treatments. As this review outlines, discovering that exosomes mediate important cell-to-cell communication through the delivery of HIV-1 and host molecules has underlined particular interest for the use of exosomes from various sources as potential HIV-1 biomarkers and therapeutic tools. However, although significant advances have been made, many questions remain about the role of exosomes in general and biofluid exosomes in particular in intercellular communication, immune modulation, and immune surveillance. Additionally, little is known about the precise molecular mechanisms of biofluid exosome biogenesis, release, targeting, and interactions with target cells within the organs where they are released. Biofluid exosomes are heterogenous vesicles derived from different cell types and, as a result, they are likely to perform specific functions in their cognate environment. This issue remains to be clarified. For biofluid exosomes to be a reliable diagnostic and therapeutic tool, robust isolation systems that will purify exosomes of interest from total EV populations must emerge. Because exosomes encase HIV-1-derived or host-derived factors, these factors constitute a network of communication that may act in a specific context or in association with many other players. The consequence is eventual conditioning of host cells. Studies that will elucidate the physiological roles of biofluid exosomes in HIV-1 infection and transmission are imperative before exosomes themselves or biofluid-derived exosome-based synthetic nanoparticles can be used diagnostically and therapeutically. Currently, many questions such as (i) which cell types release and internalize biofluid exosomes *in vivo*; (ii) what is the half-life of biofluid exosomes *in vivo*; and (iii) what is the mechanism of exosomal internalization *in vivo*, remain unanswered. Identifying the answers to these questions will fundamentally push the frontiers of our understanding of the roles exosomes play in HIV-1 pathogenesis. 

## References

[1] Harding C., Heuser J., Stahl P. (1983). Receptor-mediated endocytosis of transferrin and recycling of the transferrin receptor in rat reticulocytes. J. Cell Biol..

[2] Pan B.T., Teng K., Wu C., Adam M., Johnstone R.M. (1985). Electron microscopic evidence for externalization of the transferrin receptor in vesicular form in sheep reticulocytes. J. Cell Biol..

[3] Johnstone R.M., Adam M., Hammond J.R., Orr L., Turbide C. (1987). Vesicle formation during reticulocyte maturation. Association of plasma membrane activities with released vesicles (exosomes). J. Biol. Chem..

[4] Raposo G., Nijman H.W., Stoorvogel W., Liejendekker R., Harding C.V., Melief C.J., Geuze H.J. (1996). B lymphocytes secrete antigen-presenting vesicles. J. Exp. Med..

[5] Valadi H., Ekstrom K., Bossios A., Sjostrand M., Lee J.J., Lotvall J.O. (2007). Exosome-mediated transfer of mRNAs and microRNAs is a novel mechanism of genetic exchange between cells. Nat. Cell Biol..

[6] Simons M., Raposo G. (2009). Exosomes—Vesicular carriers for intercellular communication. Curr. Opin. Cell Biol..

[7] Bobrie A., Colombo M., Raposo G., Thery C. (2011). Exosome secretion: Molecular mechanisms and roles in immune responses. Traffic.

[8] Thery C., Ostrowski M., Segura E. (2009). Membrane vesicles as conveyors of immune responses. Nat. Rev. Immunol..

[9] Lotvall J., Valadi H. (2007). Cell to cell signalling via exosomes through esRNA. Cell Adhes. Migrat..

[10] Caby M.P., Lankar D., Vincendeau-Scherrer C., Raposo G., Bonnerot C. (2005). Exosomal-like vesicles are present in human blood plasma. Int. Immunol..

[11] Pisitkun T., Shen R.F., Knepper M.A. (2004). Identification and proteomic profiling of exosomes in human urine. Proc. Natl. Acad. Sci. USA.

[12] Palanisamy V., Sharma S., Deshpande A., Zhou H., Gimzewski J., Wong D.T. (2010). Nanostructural and transcriptomic analyses of human saliva derived exosomes. PLoS ONE.

[13] Admyre C., Johansson S.M., Qazi K.R., Filen J.J., Lahesmaa R., Norman M., Neve E.P., Scheynius A., Gabrielsson S. (2007). Exosomes with immune modulatory features are present in human breast milk. J. Immunol..

[14] Admyre C., Grunewald J., Thyberg J., Gripenback S., Tornling G., Eklund A., Scheynius A., Gabrielsson S. (2003). Exosomes with major histocompatibility complex class II and co-stimulatory molecules are present in human BAL fluid. Eur. Respir. J..

[15] Llorente A., de Marco M.C., Alonso M.A. (2004). Caveolin-1 and MAL are located on prostasomes secreted by the prostate cancer PC-3 cell line. J. Cell Sci..

[16] Sahlen G.E., Egevad L., Ahlander A., Norlen B.J., Ronquist G., Nilsson B.O. (2002). Ultrastructure of the secretion of prostasomes from benign and malignant epithelial cells in the prostate. Prostate.

[17] Ronquist G.K., Larsson A., Stavreus-Evers A., Ronquist G. (2012). Prostasomes are heterogeneous regarding size and appearance but affiliated to one DNA-containing exosome family. Prostate.

[18] Sullivan R., Saez F., Girouard J., Frenette G. (2005). Role of exosomes in sperm maturation during the transit along the male reproductive tract. Blood Cells Mol. Dis..

[19] Frenette G., Girouard J., D’Amours O., Allard N., Tessier L., Sullivan R. (2010). Characterization of two distinct populations of epididymosomes collected in the intraluminal compartment of the bovine cauda epididymis. Biol. Reprod..

[20] Renneberg H., Albrecht M., Kurek R., Krause E., Lottspeich F., Aumuller G., Wilhelm B. (2001). Identification and characterization of neutral endopeptidase (EC 3. 4. 24. 11) from human prostasomes—Localization in prostatic tissue and cell lines. Prostate.

[21] Renneberg H., Konrad L., Dammshauser I., Seitz J., Aumuller G. (1997). Immunohistochemistry of prostasomes from human semen. Prostate.

[22] Skibinski G., Kelly R.W., James K. (1994). Expression of a common secretory granule specific protein as a marker for the extracellular organelles (prostasomes) in human semen. Fertil. Steril..

[23] Stridsberg M., Fabiani R., Lukinius A., Ronquist G. (1996). Prostasomes are neuroendocrine-like vesicles in human semen. Prostate.

[24] Madison M.N., Roller R.J., Okeoma C.M. (2014). Human semen contains exosomes with potent anti-HIV-1 activity. Retrovirology.

[25] Vojtech L., Woo S., Hughes S., Levy C., Ballweber L., Sauteraud R.P., Strobl J., Westerberg K., Gottardo R., Tewari M. (2014). Exosomes in human semen carry a distinctive repertoire of small non-coding RNAs with potential regulatory functions. Nucleic Acids Res..

[26] Kaur S., Singh S.P., Elkahloun A.G., Wu W., Abu-Asab M.S., Roberts D.D. (2014). CD47-dependent immunomodulatory and angiogenic activities of extracellular vesicles produced by T cells. Matrix Biol..

[27] Madison M.N., Jones P.H., Okeoma C.M. (2015). Exosomes in human semen restrict HIV-1 transmission by vaginal cells and block intravaginal replication of LP-BM5 murine AIDS virus complex. Virology.

[28] Li J., Liu K., Liu Y., Xu Y., Zhang F., Yang H., Liu J., Pan T., Chen J., Wu M. (2013). Exosomes mediate the cell-to-cell transmission of IFN-α-induced antiviral activity. Nat. Immunol..

[29] Naslund T.I., Paquin-Proulx D., Paredes P.T., Vallhov H., Sandberg J.K., Gabrielsson S. (2014). Exosomes from breast milk inhibit HIV-1 infection of dendritic cells and subsequent viral transfer to CD4+ T cells. Aids.

[30] Arenaccio C., Chiozzini C., Columba-Cabezas S., Manfredi F., Affabris E., Baur A., Federico M. (2014). Exosomes from human immunodeficiency virus type 1 (HIV-1)-infected cells license quiescent CD4+ T lymphocytes to replicate HIV-1 through a Nef- and ADAM17-dependent mechanism. J. Virol..

[31] Arenaccio C., Chiozzini C., Columba-Cabezas S., Manfredi F., Federico M. (2014). Cell activation and HIV-1 replication in unstimulated CD4+ T lymphocytes ingesting exosomes from cells expressing defective HIV-1. Retrovirology.

[32] Mahauad-Fernandez W.D., Jones P.H., Okeoma C.M. (2014). Critical role for BST-2 in acute Chikungunya virus infection. J. Gen. Virol..

[33] De Carvalho J.V., de Castro R.O., da Silva E.Z., Silveira P.P., da Silva-Januario M.E., Arruda E., Jamur M.C., Oliver C., Aguiar R.S., daSilva L.L. (2014). Nef neutralizes the ability of exosomes from CD4+ T cells to act as decoys during HIV-1 infection. PLoS ONE.

[34] Lenassi M., Cagney G., Liao M., Vaupotic T., Bartholomeeusen K., Cheng Y., Krogan N.J., Plemenitas A., Peterlin B.M. (2010). HIV Nef is secreted in exosomes and triggers apoptosis in bystander CD4+ T cells. Traffic.

[35] Khatua A.K., Taylor H.E., Hildreth J.E., Popik W. (2010). Inhibition of LINE-1 and Alu retrotransposition by exosomes encapsidating APOBEC3G and APOBEC3F. Virology.

[36] Oliveira D.L., Nakayasu E.S., Joffe L.S., Guimaraes A.J., Sobreira T.J., Nosanchuk J.D., Cordero R.J., Frases S., Casadevall A., Almeida I.C. (2010). Characterization of yeast extracellular vesicles: Evidence for the participation of different pathways of cellular traffic in vesicle biogenesis. PLoS ONE.

[37] Lee J.C., Lee E.J., Lee J.H., Jun S.H., Choi C.W., Kim S.I., Kang S.S., Hyun S. (2012). Klebsiella pneumoniae secretes outer membrane vesicles that induce the innate immune response. FEMS Microbiol. Lett..

[38] Thery C., Boussac M., Veron P., Ricciardi-Castagnoli P., Raposo G., Garin J., Amigorena S. (2001). Proteomic analysis of dendritic cell-derived exosomes: A secreted subcellular compartment distinct from apoptotic vesicles. J. Immunol..

[39] Van Niel G., Porto-Carreiro I., Simoes S., Raposo G. (2006). Exosomes: A common pathway for a specialized function. J. Biochem..

[40] Hemler M.E. (2003). Tetraspanin proteins mediate cellular penetration, invasion, and fusion events and define a novel type of membrane microdomain. Ann. Rev. Cell Dev. Biol..

[41] Zoller M. (2009). Tetraspanins: Push and pull in suppressing and promoting metastasis. Nat. Rev. Cancer.

[42] Wubbolts R., Leckie R.S., Veenhuizen P.T., Schwarzmann G., Mobius W., Hoernschemeyer J., Slot J.W., Geuze H.J., Stoorvogel W. (2003). Proteomic and biochemical analyses of human B cell-derived exosomes. Potential implications for their function and multivesicular body formation. J. Biol. Chem..

[43] Thery C., Regnault A., Garin J., Wolfers J., Zitvogel L., Ricciardi-Castagnoli P., Raposo G., Amigorena S. (1999). Molecular characterization of dendritic cell-derived exosomes. Selective accumulation of the heat shock protein HSC73. J. Cell. Biol..

[44] Matsuo H., Chevallier J., Mayran N., le Blanc I., Ferguson C., Fauré J., Blanc N.S., Matile S., Dubochet J., Sadoul R. (2004). Role of LBPA and Alix in multivesicular liposome formation and endosome organization. Science.

[45] Bellingham S.A., Coleman B.M., Hill A.F. (2012). Small RNA deep sequencing reveals a distinct miRNA signature released in exosomes from prion-infected neuronal cells. Nucleic Acids Res..

[46] Nolte-’t Hoen E.N., Buermans H.P., Waasdorp M., Stoorvogel W., Wauben M.H., ’t Hoen P.A. (2012). Deep sequencing of RNA from immune cell-derived vesicles uncovers the selective incorporation of small non-coding RNA biotypes with potential regulatory functions. Nucleic Acids Res..

[47] ExoCarta 2015. http://exocarta.org/index.html.

[48] Vesiclepedia 2015. http://www.microvesicles.org/.

[49] Colombo M., Moita C., Van Niel G., Kowal J., Vigneron J., Benaroch P., Manel N., Moita L.F., Théry C., Raposo G. (2013). Analysis of ESCRT functions in exosome biogenesis, composition and secretion highlights the heterogeneity of extracellular vesicles. J. Cell Sci..

[50] Hoshino D., Kirkbride K., Costello K., Clark E., Sinha S., Grega-Larson N., Tyska M., Weaver A. (2013). Exosome secretion is enhanced by invadopodia and drives invasive behavior. Cell Rep..

[51] Gross J.C., Chaudhary V., Bartscherer K., Boutros M. (2012). Active Wnt proteins are secreted on exosomes. Nat. Cell Biol..

[52] Baietti M.F., Zhang Z., Mortier E., Melchior A., Degeest G., Geeraerts A., Ivarsson Y., Depoortere F., Coomans C., Vermeiren E. (2012). Syndecan-syntenin-ALIX regulates the biogenesis of exosomes. Nat. Cell Biol..

[53] Stuffers S., Sem Wegner C., Stenmark H., Brech A. (2009). Multivesicular endosome biogenesis in the absence of ESCRTs. Traffic.

[54] Trajkovic K., Hsu C., Chiantia S., Rajendran L., Wenzel D., Wieland F., Schwille P., Brugger B., Simons M. (2008). Ceramide triggers budding of exosome vesicles into multivesicular endosomes. Science.

[55] Van Niel G., Charrin S., Simoes S., Romao M., Rochin L., Saftig P., Marks M., Rubinstein E., Raposo G. (2011). The Tetraspanin CD63 Regulates ESCRT-independent and -dependent endosomal sorting during melanogenesis. Dev. Cell.

[56] Perez-Hernandez D., Gutiérrez-Vázquez C., Jorge I., López-Martín S., Ursa A., Sánchez-Madrid F., Vázquez J., Yañez-Mó M. (2013). The intracellular interactome of tetraspanin-enriched microdomains reveals their function as sorting machineries toward exosomes. J. Biol. Chem..

[57] Ostrowski M., Carmo N.B., Krumeich S., Fanget I., Raposo G., Savina A., Moita C.F., Schauer K., Hume A.N., Freitas R.P. (2010). Rab27a and Rab27b control different steps of the exosome secretion pathway. Nat. Cell Biol..

[58] Savina A., Fader C.M., Damiani M.T., Colombo M.I. (2005). Rab11 promotes docking and fusion of multivesicular bodies in a calcium-dependent manner. Traffic.

[59] Garrus J.E., Von Schwedler U.K., Pornillos O.W., Morham S.G., Zavitz K.H., Wang H.E., Wettstein D.A., Stray K.M., Côté M., Rich R.L. (2001). Tsg101 and the vacuolar protein sorting pathway are essential for HIV-1 budding. Cell.

[60] Pornillos O., Higginson D.S., Stray K.M., Fisher R.D., Garrus J.E., Payne M., He G.P., Wang H.E., Morham S.G., Sundquist W.I. (2003). HIV Gag mimics the Tsg101-recruiting activity of the human Hrs protein. J. Cell Biol..

[61] Booth A.M., Fang Y., Fallon J.K., Yang J.M., Hildreth J.E.K., Gould S.J., Sandefur S., Varthakavi V. (2006). Exosomes and HIV Gag bud from endosome-like domains of the T cell plasma membrane. J. Cell Biol..

[62] Wang J.J., Horton R., Varthakavi V., Spearman P., Ratner L. (1999). Formation and release of virus-like particles by HIV-1 matrix protein. Aids.

[63] Cantin R., Diou J., Belanger D., Tremblay A.M., Gilbert C. (2008). Discrimination between exosomes and HIV-1: Purification of both vesicles from cell-free supernatants. J. Immunol. Methods.

[64] Fang Y., Wu N., Gan X., Yan W., Morrell J.C., Gould S.J. (2007). Higher-order oligomerization targets plasma membrane proteins and HIV gag to exosomes. PLoS Biol..

[65] Usami Y., Popov S., Popova E., Inoue M., Weissenhorn W., Gottlinge H.G. (2009). The ESCRT pathway and HIV-1 budding. Biochem. Soc. Trans..

[66] Chertova E., Chertov O., Coren L.V., Roser J.D., Trubey C.M., Bess J.W., Sowder R.C., Barsov E., Hood B.L., Fisher R.J. (2006). Proteomic and biochemical analysis of purified human immunodeficiency virus type 1 produced from infected monocyte-derived macrophages. J. Virol..

[67] Gould S.J., Booth A.M., Hildreth J.E. (2003). The Trojan exosome hypothesis. Proc. Natl. Acad. Sci. USA.

[68] Grigorov B., Attuil-Audenis V., Perugi F., Nedelec M., Watson S., Pique C., Darlix J.L., Conjeaud H., Muriaux D. (2009). A role for CD81 on the late steps of HIV-1 replication in a chronically infected T cell line. Retrovirology.

[69] Izquierdo-Useros N., Naranjo-Gomez M., Archer J., Hatch S.C., Erkizia I., Blanco J., Borras F.E., Puertas M.C., Connor J.H., Fernandez-Figueras M.T. (2009). Capture and transfer of HIV-1 particles by mature dendritic cells converges with the exosome-dissemination pathway. Blood.

[70] Jolly C., Sattentau Q.J. (2007). Human immunodeficiency virus type 1 assembly, budding, and cell-cell spread in T cells take place in tetraspanin-enriched plasma membrane domains. J. Virol..

[71] Sato K., Aoki J., Misawa N., Daikoku E., Sano K., Tanaka Y., Koyanagi Y. (2008). Modulation of human immunodeficiency virus type 1 infectivity through incorporation of tetraspanin proteins. J. Virol..

[72] Izquierdo-Useros N., Lorizate M., Puertas M.C., Rodriguez-Plata M.T., Zangger N., Erikson E., Pino M., Erkizia I., Glass B., Clotet B. (2012). Siglec-1 is a novel dendritic cell receptor that mediates HIV-1 trans-infection through recognition of viral membrane gangliosides. PLoS Biol..

[73] Saunderson S.C., Dunn A.C., Crocker P.R., McLellan A.D. (2014). CD169 mediates the capture of exosomes in spleen and lymph node. Blood.

[74] Izquierdo-Useros N., Blanco J., Erkizia I., Fernandez-Figueras M.T., Borras F.E., Naranjo-Gomez M., Bofill M., Ruiz L., Clotet B., Martinez-Picado J. (2007). Maturation of blood-derived dendritic cells enhances human immunodeficiency virus type 1 capture and transmission. J. Virol..

[75] Puryear W.B., Akiyama H., Geer S.D., Ramirez N.P., Yu X., Reinhard B.M., Gummuluru S. (2013). Interferon-inducible mechanism of dendritic cell-mediated HIV-1 dissemination is dependent on Siglec-1/CD169. PLoS Pathog..

[76] Raymond A.D., Campbell-Sims T.C., Khan M., Lang M., Huang M.B., Bond V.C., Powell M.D. (2011). HIV Type 1 Nef is released from infected c ells in CD45+ microvesicles and is present in the plasma of HIV-infected individuals. AIDS Res. Hum. Retrovir..

[77] Muratori C., Cavallin L.E., Kratzel K., Tinari A., de Milito A., Fais S., D’Aloja P., Federico M., Vullo V., Fomina A. (2009). Massive secretion by T cells is caused by HIV Nef in infected cells and by Nef transfer to bystander cells. Cell Host Microbe.

[78] Ali S.A., Huang M.B., Campbell P.E., Roth W.W., Campbell T., Khan M., Newman G., Villinger F., Powell M.D., Bond V.C. (2010). Genetic characterization of HIV type 1 Nef-induced vesicle secretion. AIDS Res. Hum. Retrovir..

[79] Lubben N.B., Sahlender D.A., Motley A.M., Lehner P.J., Benaroch P., Robinson M.S. (2007). HIV-1 Nef-induced down-regulation of MHC class I requires AP-1 and clathrin but not PACS-1 and is impeded by AP-2. Mol. Biol. Cell.

[80] Schaefer M.R., Wonderlich E.R., Roeth J.F., Leonard J.A., Collins K.L. (2008). HIV-1 Nef targets MHC-I and CD4 for degradation via a final common β-COP-dependent pathway in T cells. PLoS Pathog..

[81] Da Silva L.L., Sougrat R., Burgos P.V., Janvier K., Mattera R., Bonifacino J.S. (2009). Human immunodeficiency virus type 1 Nef protein targets CD4 to the multivesicular body pathway. J. Virol..

[82] Roeth J.F., Williams M., Kasper M.R., Filzen T.M., Collins K.L. (2004). HIV-1 Nef disrupts MHC-I trafficking by recruiting AP-1 to the MHC-I cytoplasmic tail. J. Cell Biol..

[83] Anderson S.J., Lenburg M., Landau N.R., Garcia J.V. (1994). The cytoplasmic domain of CD4 is sufficient for its down-regulation from the cell surface by human immunodeficiency virus type 1 Nef. J. Virol..

[84] Rhee S.S., Marsh J.W. (1994). Human immunodeficiency virus type 1 Nef-induced down-modulation of CD4 is due to rapid internalization and degradation of surface CD4. J. Virol..

[85] Amorim N.A., da Silva E.M., de Castro R.O., da Silva-Januario M.E., Mendonca L.M., Bonifacino J.S., da Costa L.J., da Silva L.L. (2014). Interaction of HIV-1 Nef protein with the host protein Alix promotes lysosomal targeting of CD4 receptor. J. Biol. Chem..

[86] Campbell T.D., Khan M., Huang M.B., Bond V.C., Powell M.D. (2008). HIV-1 Nef protein is secreted into vesicles that can fuse with target cells and virions. Ethn. Dis..

[87] Columba Cabezas S., Federico M. (2013). Sequences within RNA coding for HIV-1 Gag p17 are efficiently targeted to exosomes. Cell Microbiol..

[88] Chivero E.T., Bhattarai N., Rydze R.T., Winters M.A., Holodniy M., Stapleton J.T. (2014). Human pegivirus RNA is found in multiple blood mononuclear cells *in vivo* and serum-derived viral RNA-containing particles are infectious *in vitro*. J. Gen. Virol..

[89] Narayanan A., Iordanskiy S., Das R., Van Duyne R., Santos S., Jaworski E., Guendel I., Sampey G., Dalby E., Iglesias-Ussel M. (2013). Exosomes derived from HIV-1-infected cells contain trans-activation response element RNA. J. Biol. Chem..

[90] Bernard M.A., Zhao H., Yue S.C., Anandaiah A., Koziel H., Tachado S.D. (2014). Novel HIV-1 miRNAs stimulate TNFalpha release in human macrophages via TLR8 signaling pathway. PLoS ONE.

[91] Kadiu I., Narayanasamy P., Dash P.K., Zhang W., Gendelman H.E. (2012). Biochemical and biologic characterization of exosomes and microvesicles as facilitators of HIV-1 infection in macrophages. J. Immunol..

[92] Mack M., Kleinschmidt A., Bruhl H., Klier C., Nelson P.J., Cihak J., Plachy J., Stangassinger M., Erfle V., Schlondorff D. (2000). Transfer of the chemokine receptor CCR5 between cells by membrane-derived microparticles: A mechanism for cellular human immunodeficiency virus 1 infection. Nat. Med..

[93] Rozmyslowicz T., Majka M., Kijowski J., Murphy S.L., Conover D.O., Poncz M., Ratajczak J., Gaulton G.N., Ratajczak M.Z. (2003). Platelet- and megakaryocyte-derived microparticles transfer CXCR4 receptor to CXCR4-null cells and make them susceptible to infection by X4-HIV. AIDS.

[94] Chariot P., Dubreuil-Lemaire M.L., Zhou J.Y., Lamia B., Dume L., Larcher B., Monnet I., Levy Y., Astier A., Gherardi R. (1997). Muscle involvement in human immunodeficiency virus-infected patients is associated with marked selenium deficiency. Muscle Nerve.

[95] Scadden D.T., Zeira M., Woon A., Wang Z., Schieve L., Ikeuchi K., Lim B., Groopman J.E. (1990). Human immunodeficiency virus infection of human bone marrow stromal fibroblasts. Blood.

[96] Permanyer M., Ballana E., Badia R., Pauls E., Clotet B., Este J.A. (2012). Trans-infection but not infection from within endosomal compartments after cell-to-cell HIV-1 transfer to CD4+ T cells. J. Biol. Chem..

[97] Peterlin B.M., Trono D. (2003). Hide, shield and strike back: How HIV-infected cells avoid immune eradication. Nat. Rev. Immunol..

[98] Costa L.J., Chen N., Lopes A., Aguiar R.S., Tanuri A., Plemenitas A., Peterlin B.M. (2006). Interactions between Nef and AIP1 proliferate multivesicular bodies and facilitate egress of HIV-1. Retrovirology.

[99] Madrid R., Janvier K., Hitchin D., Day J., Coleman S., Noviello C., Bouchet J., Benmerah A., Guatelli J., Benichou S. (2005). Nef-induced alteration of the early/recycling endosomal compartment correlates with enhancement of HIV-1 infectivity. J. Biol. Chem..

[100] Stumptner-Cuvelette P., Jouve M., Helft J., Dugast M., Glouzman A.S., Jooss K., Raposo G., Benaroch P. (2003). Human immunodeficiency virus-1 Nef expression induces intracellular accumulation of multivesicular bodies and major histocompatibility complex class II complexes: Potential role of phosphatidylinositol 3-kinase. Mol. Biol. Cell.

[101] Admyre C., Johansson S.M., Paulie S., Gabrielsson S. (2006). Direct exosome stimulation of peripheral human T cells detected by ELISPOT. Eur. J. Immunol..

[102] Kesimer M., Scull M., Brighton B., deMaria G., Burns K., O’Neal W., Pickles R.J., Sheehan J.K. (2009). Characterization of exosome-like vesicles released from human tracheobronchial ciliated epithelium: A possible role in innate defense. FASEB J..

[103] Khatua A.K., Taylor H.E., Hildreth J.E., Popik W. (2009). Exosomes packaging APOBEC3G confer human immunodeficiency virus resistance to recipient cells. J. Virol..

[104] Xiang X., Liu Y., Zhuang X., Zhang S., Michalek S., Taylor D.D., Grizzle W., Zhang H.G. (2010). TLR2-mediated expansion of MDSCs is dependent on the source of tumor exosomes. Am. J. Pathol..

[105] Carlsson L., Pahlson C., Bergquist M., Ronquist G., Stridsberg M. (2000). Antibacterial activity of human prostasomes. Prostate.

[106] Kitamura M., Namiki M., Matsumiya K., Tanaka K., Matsumoto M., Hara T., Kiyohara H., Okabe M., Okuyama A., Seya T. (1995). Membrane cofactor protein (CD46) in seminal plasma is a prostasome-bound form with complement regulatory activity and measles virus neutralizing activity. Immunology.

[107] Arienti G., Carlini E., Nicolucci A., Cosmi E.V., Santi F., Palmerini C.A. (1999). The motility of human spermatozoa as influenced by prostasomes at various pH levels. Biol. Cell.

[108] Babiker A.A., Ronquist G., Nilsson U.R., Nilsson B. (2002). Transfer of prostasomal CD59 to CD59-deficient red blood cells results in protection against complement-mediated hemolysis. Am. J. Reprod. Immunol..

[109] Burden H.P., Holmes C.H., Persad R., Whittington K. (2006). Prostasomes—Their effects on human male reproduction and fertility. Hum. Reprod. Update.

[110] Cross N.L., Mahasreshti P. (1997). Prostasome fraction of human seminal plasma prevents sperm from becoming acrosomally responsive to the agonist progesterone. Arch. Androl..

[111] Kelly R.W. (1995). Immunosuppressive mechanisms in semen: Implications for contraception. Hum. Reprod..

[112] Palmerini C.A., Saccardi C., Carlini E., Fabiani R., Arienti G. (2003). Fusion of prostasomes to human spermatozoa stimulates the acrosome reaction. Fertil. Steril..

[113] Park K.H., Kim B.J., Kang J., Nam T.S., Lim J.M., Kim H.T., Park J.K., Kim Y.G., Chae S.W., Kim U.H. (2011). Ca^2+^ signaling tools acquired from prostasomes are required for progesterone-induced sperm motility. Sci. Signal..

[114] Skibinski G., Kelly R.W., Harkiss D., James K. (1992). Immunosuppression by human seminal plasma—Extracellular organelles (prostasomes) modulate activity of phagocytic cells. Am. J. Reprod. Immunol..

[115] Tarazona R., Delgado E., Guarnizo M.C., Roncero R.G., Morgado S., Sanchez-Correa B., Gordillo J.J., Dejulian J., Casado J.G. (2011). Human prostasomes express CD48 and interfere with NK cell function. Immunobiology.

[116] Babiker A.A., Nilsson B., Ronquist G., Carlsson L., Ekdahl K.N. (2005). Transfer of functional prostasomal CD59 of metastatic prostatic cancer cell origin protects cells against complement attack. Prostate.

[117] Jones J.L., Saraswati S., Block A.S., Lichti C.F., Mahadevan M., Diekman A.B. (2010). Galectin-3 is associated with prostasomes in human semen. Glycoconj. J..

[118] Kelly R.W., Holland P., Skibinski G., Harrison C., McMillan L., Hargreave T., James K. (1991). Extracellular organelles (prostasomes) are immunosuppressive components of human semen. Clin. Exp. Immunol..

[119] Bechoua S., Rieu I., Sion B., Grizard G. (2011). Prostasomes as potential modulators of tyrosine phosphorylation in human spermatozoa. Syst. Biol. Reprod. Med..

[120] Pons-Rejraji H., Artonne C., Sion B., Brugnon F., Canis M., Janny L., Grizard G. (2011). Prostasomes: Inhibitors of capacitation and modulators of cellular signalling in human sperm. Int. J. Androl..

[121] Saez F., Motta C., Boucher D., Grizard G. (1998). Antioxidant capacity of prostasomes in human semen. Mol. Hum. Reprod..

[122] Saez F., Motta C., Boucher D., Grizard G. (2000). Prostasomes inhibit the NADPH oxidase activity of human neutrophils. Mol. Hum. Reprod..

[123] Cross N.L. (1996). Human seminal plasma prevents sperm from becoming acrosomally responsive to the agonist, progesterone: Cholesterol is the major inhibitor. Biol. Reprod..

[124] Kelly R.W., Critchley H.O. (1997). Immunomodulation by human seminal plasma: A benefit for spermatozoon and pathogen?. Hum. Reprod..

[125] Vojtech L., Hughes S., Levy C., Tewari M., Hladik F. (2014). Exosomes in human semen impair antigen-presenting cell function and decrease antigen-specific T cell responses (MUC7P.767). J. Immunol..

[126] Konadu K.A., Chu J., Huang M.B., Amancha P.K., Armstrong W., Powell M.D., Villinger F., Bond V.C. (2014). Association of Cytokines with Exosomes in the Plasma of HIV-1-Seropositive Individuals. J. Infect. Dis..

[127] Wang X., Ye L., Hou W., Zhou Y., Wang Y.J., Metzger D.S., Ho W.Z. (2009). Cellular microRNA expression correlates with susceptibility of monocytes/macrophages to HIV-1 infection. Blood.

[128] Mantri C.K., Mantri J.V., Pandhare J., Dash C. (2014). Methamphetamine inhibits HIV-1 replication in CD4+ T cells by modulating anti-HIV-1 miRNA expression. Am. J. Pathol..

[129] Hariharan M., Scaria V., Pillai B., Brahmachari S.K. (2005). Targets for human encoded microRNAs in *HIV* genes. Biochem. Biophys. Res. Commun..

[130] Ahluwalia J.K., Khan S.Z., Soni K., Rawat P., Gupta A., Hariharan M., Scaria V., Lalwani M., Pillai B., Mitra D. (2008). Human cellular microRNA hsa-miR-29a interferes with viral nef protein expression and HIV-1 replication. Retrovirology.

[131] Mantri C.K., Pandhare Dash J., Mantri J.V., Dash C.C. (2012). Cocaine enhances HIV-1 replication in CD4+ T cells by down-regulating miR-125b. PLoS ONE.

[132] Sung T.L., Rice A.P. (2009). miR-198 inhibits HIV-1 gene expression and replication in monocytes and its mechanism of action appears to involve repression of cyclin T1. PLoS Pathog..

[133] Chevillet J.R., Kang Q., Ruf I.K., Briggs H.A., Vojtech L.N., Hughes S.M., Cheng H.H., Arroyo J.D., Meredith E.K., Gallichotte E.N. (2014). Quantitative and stoichiometric analysis of the microRNA content of exosomes. Proc. Natl. Acad. Sci. USA.

[134] Mittelbrunn M., Gutierrez-Vazquez C., Villarroya-Beltri C., Gonzalez S., Sanchez-Cabo F., Gonzalez M.A., Bernad A., Sanchez-Madrid F. (2011). Unidirectional transfer of microRNA-loaded exosomes from T cells to antigen-presenting cells. Nat. Commun..

[135] Conde-Vancells J., Rodriguez-Suarez E., Gonzalez E., Berisa A., Gil D., Embade N., Valle M., Luka Z., Elortza F., Wagner C. (2010). Candidate biomarkers in exosome-like vesicles purified from rat and mouse urine samples. Proteomics.

